# Genetic Selection Approach for Semen Characteristics in Thai Native Grandparent Roosters (Pradu Hang Dum) Using Random Regression Test-Day Models and Selection Indices

**DOI:** 10.3390/ani14131881

**Published:** 2024-06-26

**Authors:** Iin Mulyawati Daryatmo, Jiraporn Juiputta, Vibuntita Chankitisakul, Wuttigrai Boonkum

**Affiliations:** 1 Department of Animal Science, Faculty of Agriculture, Khon Kean University, Khon Kean 40002, Thailand; iin.d@kkumail.com (I.M.D.); jiraporn.ju@kkumail.com (J.J.); vibuch@kku.ac.th (V.C.); 2 Network Center for Animal Breeding and Omics Research, Khon Kaen University, Khon Kaen 40002, Thailand

**Keywords:** breeding value, heritability, genetic parameter, indigenous chicken

## Abstract

**Simple Summary:**

Semen characteristics are directly related to the success of the reproductive system and reflect the production efficiency of native chickens. Genetic evaluation using quantitative genetics is one of the highly effective methods for increasing selection accuracy; however, determining the appropriate genetic model is one of the cornerstones of this success. Variability of estimates over the period of the semen collection obtained through RRM could be useful to further refine the breeding program for selection, deciding the production and culling period.

**Abstract:**

The aim of this study was to analyze suitable genetic models and selection indices to estimate the genetic parameters and breeding values of native Thai roosters. A total of 3475 records of seven semen traits (mass movement, semen pH, semen color, volume, sperm viability, sperm abnormalities, and sperm concentration) from 242 Thai native grandparent roosters were analyzed. Multiple-trait random regression test-day models with five covariance functions were used to analyze the variance components, genetic parameters, and breeding values. The selection index (SI) was calculated to determine the optimal genetic value for different selection percentages. The results showed that a multiple-trait random regression test-day model with a second-order Legendre polynomial function was the most appropriate genetic model for this population. The estimated heritability values were low to moderate, ranging from 0.110 to 0.112 (mass movement), 0.040 to 0.051 (semen pH), 0.092 to 0.097 (semen color), 0.220 to 0.225 (semen volume), 0.067 to 0.083 (sperm viability), 0.086 to 0.099 (sperm abnormalities), and 0.134 to 0.138 (sperm concentration). The repeatability values exceeded the heritability values and were within the range of 0.133 to 0.688. The genetic correlations among semen traits ranged from −0.332 to 0.677, and phenotypic correlations ranged from −0.260 to 0.460. When considering heritability and genetic correlation values, semen volume, sperm concentration, and mass movement were the top three priority semen traits calculated as selection indices. Finally, the top 10% of the selection index was recommended for creating the next generation. Our findings provide useful information on genetic parameters and an appropriate selection index of semen traits for selecting the genetics of individual Thai native grandparent roosters. The heritability estimates for semen traits reported here suggest an adequate response to selection through a genetic evaluation approach. Our results indicate that it is possible to select grandparent roosters with better reproductive performance.

## 1. Introduction

With economic growth over the last 50 years, animal-derived food consumption has increased dramatically worldwide. According to OECD-FAO statistical data for 2022, the global protein availability of poultry meat is projected to increase by 16% by 2031 [1]. Global consumption of poultry meat is projected to increase to 154 Mt over the projected period, accounting for nearly half of the additional meat consumed [1]. Consumers choose poultry because of its lower price, product consistency, adaptability, and higher protein and lower fat content. Asian countries play an important role as the world’s poultry canter, with 70% of the world’s poultry population, and have become significant broiler meat exporters to the global market.

Concurrently with commercial chickens, the production and consumption of native chickens in Thailand have increased rapidly over the past few decades because of their unique characteristics and taste, lower fat content, and high-quality protein content [2,3]. Even though native chickens are easily adaptable to tropical climates without considerable loss in production and have a lower mortality rate than commercial breed chickens [4], their lower growth performance than commercial breeds reduces competitiveness [5,6]. Thus, previous studies have focused on genetic improvements of Thai native breeds in terms of their growth performance and carcass characteristics [7], egg production [8,9], and female reproduction [10]. Meanwhile, reproductive quality has continuously decreased because semen traits received less attention [11]. Sperm quality characteristics, such as volume, semen color, pH, sperm concentration, sperm motility, sperm viability, and sperm abnormality, are routinely assessed in breeding roosters to indicate fertility potential or sperm quality after preservation. Moreover, the relationship between these parameters serves as an excellent indicator of reproductive potential and aids in determining the fertility and hatchability of eggs [12,13]. Various reports document the relationship between different sperm quality traits. For instance, chickens producing higher semen volumes exhibit better sperm quality in terms of sperm motility and concentration compared to those with lower semen volumes [14]. High sperm concentration and volume ensure a sufficient number of viable sperm are available for insemination [15,16]. Additionally, semen viability traits can reflect the physiological status and serve as measures of interactions with semen during collection, preservation, and insemination processes. The correlation between semen pH and sperm quality characteristics, such as motility, viability, concentration, and volume, is well-established; an acidic environment can directly impact sperm quality [17]. Sperm morphology is indicative of the physiological or pathological status of the male in sperm production and storage in extragonadal ducts. In addition, sperm abnormalities and pH levels impact viability and motility, with optimal pH supporting better sperm health and fewer abnormalities [18]. Semen color is directly associated with sperm concentration; however, it is reported that variations in semen color may arise partly due to contaminants, such as urine, feces, blood, or low sperm concentration [19].

Random regression models (RRMs) [20] facilitate the genetic improvement of livestock such as dairy cattle, sheep, and poultry [21,22,23]. RRMs make it possible to describe characteristics across ages and apply more precise estimates of genetic traits in a selection based on age [23]. Especially in poultry, several studies have succeeded in using RRMs for genetic improvement of fertility and hatchability to increase egg production in poultry breeding programs [21,23]; however, few studies examined semen characteristics in poultry, especially about native chickens, and most of them estimate genetic parameters [24,25,26], which cannot be used for individual genetic selection yet. Individual genetic selection of semen traits plays a crucial role in modern livestock production systems by enabling farmers to breed animals with desired characteristics, leading to improved efficiency, productivity, and sustainability in agriculture. Therefore, this study was conducted to analyze and identify an appropriate RRM with covariance functions for improved individual genetic selection of semen traits in Thai native chickens. In addition to offering significant insights into poultry breeding methodologies, the finding of this study may serve as a definitive guideline for the strategic planning of genetic selection, especially in the grandparent generation, which will be of considerable value for practical implementation.

## 2. Materials and Methods

### 2.1. Animal Ethics and Animal Management

The Institutional Animal Care and Use Committee of Khon Kaen University reviewed and approved this study based on the Ethics of Animal Experimentation of the National Research Council of Thailand (No. IACUC-KKU-114/66, 6 October 2023). This study was conducted at the experimental farm of the Network Center for Animal Breeding and Omics Research, Faculty of Agriculture, Khon Kaen University, Thailand. A total of 3475 records of 242 Thai native grandparent roosters (Pradu Hang Dum) aged 1–4 years were housed individually in 45 × 50 × 60 cm battery cages and raised in an open-house system exposed to sunlight and natural ambient temperature. Approximately 110 g feed/bird/day, consisting of commercial breeder feed for roosters (90.07% dry matter, 17.15% crude protein, 3.35% crude fiber, 3.99% ether extract, and 9.75% ash) was provided along with ad libitum access to drinking during the experimental period. Additional data collected included the animal identification (ID) of the roosters, body weight, age, ambient temperature and humidity index, month and year of birth, and month and year of semen data collection.

### 2.2. Semen Collection and Evaluation

Semen samples were collected once per week (each Saturday) for 52 weeks using the dorsal-abdominal massage method [27]; semen was carefully placed in a 1.5-mL Eppendorf tube containing 0.1 mL IGGKPh diluent. The semen samples were protected from light and stored at 22–25 °C during transport to the laboratory within 20 min of collection for standard semen analysis procedures, including macroscopic and microscopic evaluation. Semen collection was always performed by the same person to maximize quality and quantity, and the semen was carefully handled to prevent cross-contamination during semen collection.

Semen volume was recorded using a graded 1-mL syringe. Semen color was assessed subjectively using a scoring scale modified from Zemjanis [28] as follows: 5, milky white; 4, creamy; 3, transparent; 2, yellow; and 1, bloodshot. A pH meter (HANNA HI98103) was used to determine the pH.

Sperm motility was classified as a mass movement. For this, one drop of semen sample was placed on a slide without a coverslip, examined under a light microscope at 400× magnification (Olympus CH30, Tokyo, Japan), and scored on a 1–5-point scaling system according to the methods of Peters et al. [29]. Sperm viability and sperm abnormality were assessed simultaneously through eosin-nigrosine staining [30]. Briefly, a 5-µL drop of fresh semen was placed on a slide, and 20 µL eosin-nigrosine was added, followed by gentle mixing. The mixture was left to dry (using an air dryer) for a few minutes. At least 300 sperm were counted under a light microscope at 1000× magnification to determine the proportion of live sperm. Stained sperm were considered dead sperm, whereas non-stained sperm were considered live sperm, and the results were expressed as percentages. Meanwhile, sperm abnormalities determine whether one has morphological anomalies (such as anomalies in the head, tail, connecting piece, or terminal piece). The sperm concentration was determined using a hemocytometer chamber. Five µL of semen sample was diluted with 195 µL of sodium chloride. The diluted sample was loaded into the hemocytometer and counted under a light microscope at 400× magnification. Sperm concentration was expressed as sperm × 10^9^ sperm/mL [14].

### 2.3. Genetic Model and Statistical Analysis

Data collected on the experimental farms were confirmed before genetic analysis using the Proc UNIVARIATE procedure in SAS v.9.0 software to examine data distribution, including normality, homogeneity of variance, and outliers (±3 standard deviations were defined as outlier). The variance components and genetic parameters (heritability, repeatability, genetic correlation, and phenotypic correlations) were estimated using a multiple-trait random regression test-day model with five covariance functions (Wilmink, Koops, and Grossman; second, third, and fourth order Legendre polynomial functions [LG2, LG3, and LG4, respectively]), and the average information expectation maximization restricted maximum likelihood approaches. The estimated breeding values (EBV) were analyzed using the BLUPF90 family program [31]. The model used for the analysis was as follows:

$$y_{ijklm}={HMY}_{i}+{AGE}_{j}+{BW}_{k}+\sum_{m=0}^{q}a_{lm}Z_{m\left(t\right)}+\sum_{m=0}^{q}p_{lm}Z_{m\left(t\right)}+e_{ijklm}$$

where $y_{ijklm}$ is the observation value of test-day semen traits per time, ${HMY}_{i}$ is the fixed effect of the combination of chicken hatch set and test month-year, ${AGE}_{j}$ is the fixed effect of age of roosters, ${BW}_{k}$ is the fixed effect of body weight of roosters, $a_{lm}$ is the random regression coefficient for additive genetic effects of roosters l, $p_{lm}$ is the random regression coefficient for permanent environmental effects of roosters l, $e_{ijklm}$ is the random residual effect for each observation, $Z_{m(t)}$ is the value of the coefficients of covariance functions at the test-day semen collection period t, and q is the number of coefficients of covariance functions. The number of coefficients of the covariance functions was equally well designed for additive genetic and permanent environmental effects, which depend on the number of orders. The covariance functions are the Wilmink function (WM), Koops and Grossman function (KG), and LG2, LG3, and LG4. The covariance matrix for all models as follow:

$$\mathrm{Var}\left[\begin{array}{c} a\\ p\\ e \end{array}\right]=\left[\begin{array}{ccc} G⨂A & 0 & 0\\ 0 & P⨂I & 0\\ 0 & 0 & R \end{array}\right]$$

where G and P are the covariance matrices for additive genetic and permanent environmental effects, respectively; A is the additive genetic relationship matrix among animals; I is an identity matrix; ⨂ is Kronecker product between matrices; and R is the diagonal matrix of residual variances allowed to differ for test-day semen collection. The covariance functions used in the analysis were as follows:

Wilmink function (WM; Wilmink [32]):
WM: ${f\left(t\right)=a_{0}+a_{1}t/12+a_{2}e}^{-0.05t/12}$,
where $a_{0}=1,a_{1},a_{2}=$ regression coefficient; t = months in semen collection
KG function (Koops and Grossman [33]):
KG: $f\left(t\right)=D/(1+\left({ae}^{-bt}+ct)\right)$,
where D = number of months of semen collection (12 months); t = months of semen collection at months 1, 2, 3, …, 12; a,b,c = regression coefficients.
Second, third, and fourth orders Legendre polynomial functions (LG2, LG3, LG4; Gengler et al. [34]):
LG2: $f\left(t\right)=L_{1}+L_{2}+L_{3}$
LG3: $f\left(t\right)=L_{1}+L_{2}+L_{3}+L_{4}$
LG4: $f\left(t\right)=L_{1}+L_{2}+L_{3}+L_{4}+L_{5}$,
where $L_{1}=1$, $L_{2}=\sqrt{3}L$, $L_{3}=\sqrt{\frac{5}{4}}(3L^{2}-1)$, $L_{4}=\sqrt{\frac{7}{4}}(5L^{3}-3L)$, $L_{5}=\sqrt{\frac{9}{64}}(35L^{4}-30L^{2}+3)$, $L=-1+2\frac{\left({t-t}_{min}\right)}{\left(t_{max}-t_{min}\right)}$,
where t = current month in semen data collection, $t_{min}$ = the first month in semen data collection, $t_{max}$ = the last months of semen data collection, respectively.

The best fit of a random regression test-day model with a covariance function was considered using two statistical criteria: the lowest minus twice the logarithm of the likelihood (–2logL) and Akaike’s information criterion (AIC). Estimating genetic variance and covariance on the period (t) of semen collection can be calculated from the following equations:

$$\sigma_{a_{tt}}^{2}=Z_{t}^{\prime }GZ_{t}$$

and

$$\sigma_{a_{tt+1}}=Z_{t}^{\prime }GZ_{t+1}$$

where Z = vector of each covariance function and G = the variance and covariance matrices for additive genetic effects. The EBV for animal genetic random regression coefficients was used to estimate breeding values for semen traits and was calculated as a selection indices (SI). The semen traits used to calculate the SI were determined using the three highest heritability values and the genetic correlation between the characteristics. The relative economic value (v) for each trait was calculated as the proportion of the standardized economic value to the total economic importance of all traits evaluated in the given production system. The SI equation was as follows:

$$SI=\left(v_{1}\times {EBV}_{trait1}\right)+\left(v_{2}\times {EBV}_{trait2}\right)+,\ldots ,+\left(v_{3}\times {EBV}_{trait3}\right)$$

where SI is the selection indices, $v_{1},v_{2},v_{3}$ are the relative economic values for semen traits, and ${EBV}_{trait1},\;{EBV}_{trait2},\;{EBV}_{trait3}$ are the estimated breeding values for semen traits.

## 3. Results

### 3.1. Descriptive Statistics of Semen Traits

The means ± SD of mass movement, semen pH, semen color, semen volume, sperm viability, sperm abnormalities, and sperm concentration in Thai native roosters were 3.45 ± 1.22, 6.77 ± 0.36, 4.11 ± 1.03, 0.39 ± 0.20, 93.13 ± 7.71, 8.94 ± 5.34, and 3.44 ± 1.58, respectively (Table 1). The values of all semen traits were low during the summer months in Thailand, from March to May, but were high during the winter months, from November to January. Roosters with low semen values stopped producing semen later, which applied to 10% of all roosters used in this study.

### 3.2. Selection of the Optimum Model and Genetic Parameters

The results of the model comparisons and genetic parameters are presented in Table 2. Using the test-day RRM with LG2 produced the lowest −2logL (−2870) and AIC (−2684) values for all semen traits, which explains why in estimating the genetic parameters of semen characteristics of Thai native grandparent roosters, the RRM should be used with LG2 in estimates that are the most accurate and fit with this dataset. In contrast, the RRM with LG4 showed the highest −2logL, and the AIC values precluded it for this dataset. The estimated heritability values for the semen traits were 0.110–0.112, 0.040–0.051, 0.092–0.097, 0.220–0.225, 0.067–0.083, 0.086–0.099, and 0.134–0.138 for mass movement, semen pH, semen color, semen volume, sperm viability, sperm abnormalities, and sperm concentration, respectively. The estimated repeatability values were higher than heritability values and within the ranges of 0.348–0.351, 0.212–0.255, 0.275–0.284, 0.647–0.688, 0.133–0.167, 0.172–0.198, and 0.473–0.487 for mass movement, semen pH, semen color, semen volume, sperm viability, sperm abnormalities, and sperm concentration, respectively.

**Figure 1:**
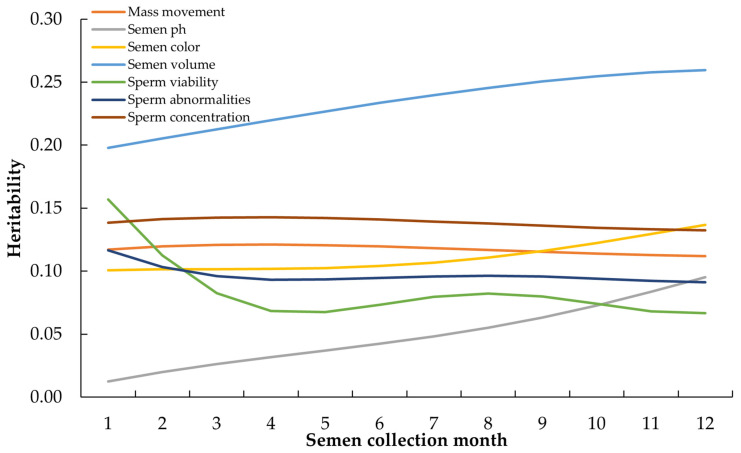
Estimation of heritability of test-day semen traits in Thai native grandparent roosters using random regression models with second orders Legendre polynomials function.

**Figure 2:**
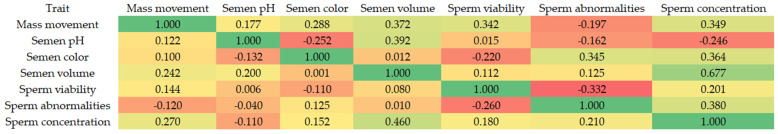
Genetic correlations (above diagonal) and phenotypic correlations (below diagonal) of test-day semen traits in Thai native grandparent roosters by correlation heatmap.

Figure 1 shows a graph of the heritability estimates throughout the semen collection period. The heritability estimates tended to increase after the first two months of semen collection for the semen volume, pH, and color. In contrast, sperm abnormalities and sperm viability tended to decrease after two months of semen collection, while mass movement and sperm concentration remained constant throughout the study period. Genetic and phenotypic correlations between semen traits are presented in Figure 2. The genetic correlations among semen traits were negative to positive and ranged from −0.332 to 0.667. Moderate positive genetic correlations were found between semen volume and sperm concentration (0.677), mass movement and sperm concentration (0.349), and mass movement and semen volume (0.372). Other genetic correlations in the semen traits were lower than 0.300, and some genetic correlations were negative, such as mass movement with sperm abnormalities (−0.197), semen pH with semen color (−0.252), semen pH with sperm abnormalities (−0.162), semen pH with sperm concentration (−0.246), semen color with sperm viability (−0.220), and sperm viability with sperm abnormalities (−0.332). The phenotypic correlations between semen traits were lower than the genetic correlations and ranged from −0.260 to 0.460; these phenotypic correlations showed the same directions as the genetic correlations.

### 3.3. Selection Indices

**Figure 3:**
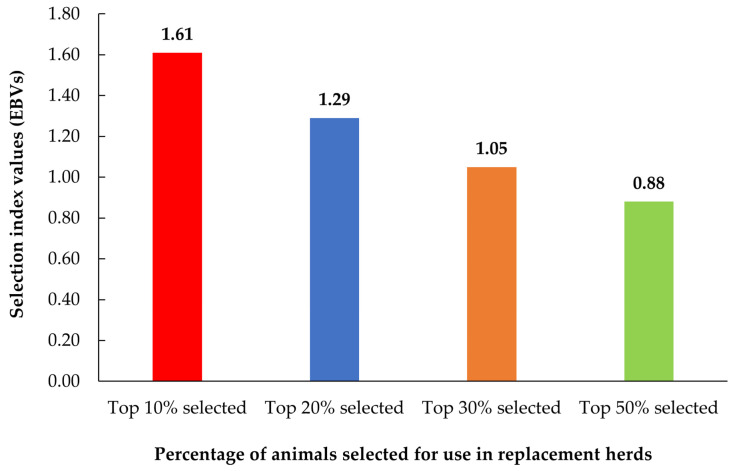
Top 10%, 20%, 30%, and 50% of the selection index values from three semen traits (semen volume, sperm concentration, mass movement) in Thai native roosters.

The top 10%, 20%, 30%, and 50% SI values for Thai native roosters are shown in Figure 3. The estimated breeding values (${EBV}_{trait1},{EBV}_{trait2},{EBV}_{trait3}$) were used to calculate the SI values. The relative economic value ($v_{1},v_{2},v_{3})$ for each semen trait was calculated as the proportion of the standardized economic value to the total economic importance of all traits evaluated in the given reproductive system. Both semen volume and sperm concentration traits were of equal importance; moreover, a moderate positive genetic correlation occurred, and therefore, the relative economic values were defined as 0.4 for semen volume and 0.4 for sperm concentration traits. For mass movement, the relative economic value was defined as 0.2. This was based on the heritability value being lower than the semen volume and sperm concentration and the genetic correlation values between the three semen traits. The SI equation is as follows: $SI=\left(0.4\times {EBV}_{Semen\;volume}\right)+\left(0.4\times {EBV}_{Sperm\;concentration}\right)+\left(0.2\times {EBV}_{Mass\;movement}\right)$. Furthermore, the percentage of animals selected for the replacement flock showed that the top 10% (1.61) had the highest SI values compared to the top 20% (1.29), top 30% (1.05), and top 50% (0.88).

## 4. Discussion

Over several decades, genetic selection has focused mainly on production traits such as growth rate, feed efficiency, and meat yield for consumption. This compromises the reproductive performance of the poultry. Therefore, genetic improvement of reproductive traits is required in the poultry industry. Semen characteristics are fundamental for the future genetic improvement of animals, as they directly influence reproductive performance, genetic diversity, economic profitability, and adaptability. Incorporating semen quality evaluations into breeding programs enables breeders to make informed decisions that optimize genetic progress and sustainability in animal production systems.

The quantity and quality of semen are essential to achieve high fertility. Our results on sperm characteristics are in agreement with those reported by Garner and Hafez [35]. The semen volume was approximately 0.39 ± 0.20 mL, which is more or less that of other breeds in previous studies [25,29,36]. Variations among chickens may be attributed to male factors in terms of body size [37]; however, in Thai native chickens, roosters produce a high semen volume (exceeding 0.3 mL) and have better sperm quality in terms of sperm motility and sperm concentration than roosters producing a low semen volume (less than 0.3 mL) [14]. Semen color is directly associated with sperm concentration. In addition, greater semen volume increases sperm fluidity, facilitating sperm movement [29] and significantly affecting fertility. The relationship between semen volume, sperm motility, and sperm concentration may indicate that a high volume determines successful fertility. Meanwhile, the sperm viability and pH of semen, which determine successful fertility, are not affected by breed [14,29] but may be affected by chicken age, nutrition, environmental factors, management practices, stress, hormonal factors, and seasonal influences, which could be controlled through management [14].

The heritability values (0.040–0.225) for all semen traits in Table 2 indicate that a low to moderate proportion of the observed variation was due to genetic factors, whereas the remaining variation was influenced by environmental factors [38]. Genetic selection can be achieved using traditional methods. The heritability values in this study were in agreement with those reported previously. For example, the heritability of seven semen traits (semen volume, pH, color, viability, motility, deformities, and concentration) in Beijing-You chickens from China at 43 weeks of age ranged from 0.03 to 0.85 [25]; in sperm motility and sperm count of White Leghorns at 36 weeks of age were 0.08 and 0.13, respectively [26]; and heritability estimates of 0.27, 0.34 and 0.26 for semen volume, sperm concentration, and sperm motility were observed in White Leghorn roosters at 26 weeks of age [24]. However, the differences in the reported estimates are probably due to age, strain, breeding methods, genetic methods, ambient temperature and relative humidity, and sample size in terms of accurate, precise, and unbiased estimates of genetic parameters and values [39]. Heritability values help breeders identify traits that have a strong genetic basis. Semen volume, sperm concentration, and mass movement showed the highest heritability values among the first three semen characteristics in the current study. Semen traits with high heritability are more likely to be passed from generation to generation. In addition, it had a relatively consistent expression value when considering heritability values over one year (Figure 1). Therefore, it is suitable for selection throughout the semen production period of male chickens. Further, breeders can focus on selecting animals that exhibit desirable traits with high heritability, leading to faster and more effective genetic improvements [38]. In summary, heritability values are essential in animal breeding to guide breeders in selecting the most effective strategies to achieve genetic improvement in desired traits within a population.

The repeatability values ranged from 0.133 to 0.688. Repeatability values < 0.5 indicate that only a small portion of the observed variation in the trait is due to permanent factors, while the majority is influenced by temporary or environmental factors; by contrast, repeatability values > 0.5 mean that the observed variation is due to permanent factors, making the trait more predictable and reliable for breeding objectives. Repeatability values contribute to the accuracy of the EBVs and selection indices. More accurate EBVs can better inform breeding decisions, resulting in higher genetic gains over time, as the selection process is based on reliable and repeatable information [40]. Repeatability values aid the efficient allocation of resources within breeding programs. Breeders can focus on fewer measurements or observations without sacrificing accuracy for traits with high repeatability, allowing for more streamlined and cost-effective breeding practices.

Genetic correlations are valuable tools for animal breeders as they provide insight into the relationships between different traits within a population. These correlations indicate how closely genetic factors influencing one trait are related to those influencing another. In this study, genetic correlation values greater than 0.4 occurred in the genetic correlations between semen volume with sperm concentration (0.677), mass movement vs. sperm concentration (0.349), and mass movement vs. semen volume (0.372). This is consistent with the results of previous studies reporting genetic correlations of semen volume with sperm concentration of 0.68 in Beijing-You chickens [25], 0.47 in frizzle chickens in Nigeria [41], and 0.16 to 0.65 in White Leghorn [24]. The genetic correlation of mass movement with sperm concentration in White Leghorns is 0.56 [26], 0.99 in Betong chickens [42], and 0.51 in White Rock roosters at 6–7 months of age [43], but −0.04 in White Leghorn roosters [25], while genetic correlations between mass movement vs. semen volume were different from those in previous studies on Beijing-You chickens (−0.02) [25], Betong chickens (−0.34) [42], and White Leghorn Strain (−0.11 to −0.15) [24]. These genetic correlations suggest a significant relationship between the genetic factors influencing the two traits. Thus, individuals with favorable genetic variants for one trait are likely to have favorable genetic variants for another trait. Consequently, improvements in one trait through selective breeding will likely result in improved correlated traits without direct selection. With a moderate-to-strong positive genetic correlation, breeders can design more efficient breeding strategies, such as selection indices or multi-trait selection, to improve both traits simultaneously. This can lead to faster and more effective genetic improvements in breeding populations. Regarding low positive genetic correlations (<0.200) among semen traits, there is a weak tendency for these traits to be inherited together. Therefore, genetic selection for traits with low and positive genetic correlations can be challenging because the relationship between traits is not very strong; however, some strategies can be employed, such as independent genetic trait selection and other genetic approaches, such as marker-assisted selection (MAS) and genomic selection.

In contrast, negative values were found for the genetic correlations between mass movement with sperm abnormalities (−0.197), semen pH with semen color (−0.252), semen pH with sperm abnormalities (−0.162), semen pH with sperm concentration (−0.246), semen color with sperm viability (−0.220), and sperm viability with sperm abnormalities (−0.332). Negative genetic correlations imply that improving one trait through selective breeding will likely result in a decrease in the other trait. Different sets of genetic factors or pathways influence such trait pairs, leading to antagonistic relationships. Genetic selection, rather than focusing solely on the trait of interest, incorporates selection for other traits with a positive genetic correlation. This approach aims to balance improvements in multiple traits simultaneously. Additionally, an index combining multiple traits was developed to produce a single selection criterion and weighted according to economic importance and genetic correlations. This allows breeders to improve several traits simultaneously without neglecting negative correlations.

The low phenotypic correlations in the present study (−0.060 to 0.270) may indicate that the two traits are genetically or environmentally independent of each other. This implies that the genetic factors influencing one trait are not strongly related to the genetic factors influencing the other or that environmental factors affecting one trait do not strongly influence the other. As a step forward, the genetic basis of these various traits should be explored, and whether there is a genetic relationship despite low phenotypic correlations should be determined. This can be achieved through genetic analyses, such as heritability estimation. Quantitative trait locus mapping or genome analysis of genetic relationships can provide insights into genetic architecture and the potential for genetic improvement. An SI is a tool used in poultry breeding to combine multiple traits into a single value to facilitate the simultaneous improvement of several characteristics. Statistical approaches allow breeders to make more informed and balanced selection decisions and meet diverse breeding objectives. The genetic correlation and heritability analyses suggest that semen volume, sperm concentration, and mass movement are the preferable representative parameters for genetic selection compared with the others. Therefore, in this study, we selected these three parameters to calculate the SI, and the results confirmed that selecting the top 10% of the flock was preferable, as the SI values were the highest compared with other proportions (Figure 3). This is in accordance with the findings of Khazraji et al. [44], who stated that selecting breeding animals from a small population resulted in higher inbreeding and decreased genetic variation; however, genetic progress increased. The SI values will be lower if many animals are selected, which is directly related to a decrease in genetic progress [45]. Therefore, we suggest that intensive selection of the top 10% of flocks with the highest genetic merit is required to obtain results precisely and timely, which would be advantageous for genetic improvement by choosing animals to align closely with the desired traits for the next generation in a short interval.

Determining the relative economic value of each semen characteristic relative economic value in a selection index is a crucial component in breeding programs by helping to balance multiple traits according to their economic importance. Given these values, breeders can make decisions based on several factors, including the heritability value of the traits, genetic correlations between traits, and the economic importance of traits that increase overall profitability and efficiency in breeding programs. To understand the economic configuration guidelines of this study, we explain why the relative economic values were defined as 0.4 for semen volume and sperm concentration traits and 0.2 for mass movement, as follows: Considering the heritability rate, it was found that the heritability of semen volume is twice that of mass movement. Therefore, the economic value we assign to semen volume is approximately twice that of mass movement. However, this principle may not apply to the economic value of sperm concentration. Thus, we also consider correlation values. The genetic correlation between semen volume and sperm concentration was 0.677, while the genetic correlation between mass movement and semen volume, as well as mass movement and sperm concentration, were 0.372 and 0.349, respectively. This indicates that the genetic relationship between semen volume and sperm concentration is almost twice as high as that between mass movement and the other traits. In other words, this higher correlation highlights the importance of both semen volume and sperm concentration. Therefore, we assign the same economic value to these two characteristics. The economic importance of traits for general poultry breeders and producers, focusing on improving sperm concentration, should be the primary goal, followed by ensuring adequate semen volume and monitoring mass movement for quality control. Optimally balancing these characteristics leads to higher fertilization success rates, greater productivity, and better economic outcomes in poultry operations.

In this study, we achieved promising results and established guidelines applicable to various poultry species in each region. However, it is important to acknowledge certain limitations that could affect the accuracy of our estimates. Key considerations include the complexity of semen quality traits, which comprise multiple components (e.g., motility, morphology) contributing to overall fertility. Unraveling the genetic basis of these traits and understanding their interactions can be particularly challenging, especially in indigenous breeds where trait expression may vary. Moreover, successful implementation of selective breeding programs necessitates adequate infrastructure and resources, such as facilities for semen collection, storage, and artificial insemination. Insufficient infrastructure may impede the practical application of genetic evaluations and selection based on semen quality traits. Finally, semen quality traits are significantly influenced by environmental factors such as nutrition, management practices, seasonality, and health status. Variations in these factors can confound genetic estimates if not properly controlled or accounted for in the analysis. Therefore, addressing these complexities is crucial for enhancing the reliability and applicability of our findings across poultry breeding programs.

## 5. Conclusions

The random regression test-day model with a second order Legendre polynomial function was the most appropriate genetic model and can be effectively used in selecting and planning animal breeding methods for semen traits in native Thai grandparent roosters. Additionally, our study found that when selecting roosters with good genetics for semen characteristics, emphasis should be placed on semen volume, sperm concentration, and mass movement as the first three characteristics and that selecting the top 10% is sufficient to preserve the excellent genetics of the grandparent generation.

## Figures and Tables

**Table 1:** Parameters of Thai native grandparent roosters and semen.

Factor	N	Mean	SD	Min	Max
Animal with records	242				
Animal with pedigrees	538				
Number of records	3475				
Mass movement (score)	3475	3.45	1.22	1.00	5.00
Semen pH (score)	3475	6.77	0.36	5.16	9.19
Semen color (score)	3475	4.11	1.03	1.00	5.00
Semen volume (mL/ejaculation)	3475	0.39	0.20	0.10	2.50
Sperm viability (%)	3475	93.13	7.71	9.46	99.71
Sperm abnormalities (%)	3475	8.94	5.34	0.92	83.64
Sperm concentration (×10^9^ sperm/mL)	3475	3.44	1.58	0.20	9.48

**Table 2:** Estimated variance components, heritability, and comparison of statistics criteria of random regression models with various covariance functions for test-day semen traits in Thai native grandparent roosters.

Trait	Model		Variance Components/Heritability/Statistic Criteria					
		$\sigma_{a}^{2}$	$\sigma_{pe}^{2}$	$\sigma_{e}^{2}$	$h^{2}$ (±SE)	t (±SE)	−2logL	AIC
Mass movement	KG	0.164	0.355	0.968	0.110 ± 0.02	0.349 ± 0.03	−2505	−2415
	WM	0.164	0.355	0.969	0.110 ± 0.02	0.349 ± 0.03	−2492	−2402
	LG2	0.164	0.352	0.968	0.111 ± 0.02	0.348 ± 0.03	−2870	−2684
	LG3	0.166	0.356	0.964	0.112 ± 0.02	0.351 ± 0.03	−2431	−2113
	LG4	0.163	0.356	0.964	0.110 ± 0.02	0.350 ± 0.03	−2088	−1602
Semen pH	KG	0.004	0.017	0.078	0.040 ± 0.01	0.212 ± 0.03	−2505	−2415
	WM	0.004	0.018	0.078	0.040 ± 0.01	0.220 ± 0.03	−2492	−2402
	LG2	0.004	0.016	0.074	0.043 ± 0.01	0.213 ± 0.03	−2870	−2684
	LG3	0.005	0.019	0.074	0.051 ± 0.01	0.245 ± 0.03	−2431	−2113
	LG4	0.005	0.020	0.073	0.051 ± 0.01	0.255 ± 0.03	−2088	−1602
Semen color	KG	0.016	0.033	0.124	0.092 ± 0.02	0.283 ± 0.03	−2505	−2415
	WM	0.016	0.033	0.124	0.092 ± 0.02	0.283 ± 0.03	−2492	−2402
	LG2	0.016	0.031	0.124	0.094 ± 0.02	0.275 ± 0.03	−2870	−2684
	LG3	0.017	0.032	0.126	0.097 ± 0.02	0.280 ± 0.03	−2431	−2113
	LG4	0.017	0.033	0.126	0.097 ± 0.02	0.284 ± 0.03	−2088	−1602
Semen volume	KG	0.018	0.037	0.025	0.225 ± 0.03	0.688 ± 0.06	−2505	−2415
	WM	0.018	0.037	0.025	0.225 ± 0.03	0.688 ± 0.06	−2492	−2402
	LG2	0.018	0.036	0.027	0.222 ± 0.03	0.667 ± 0.04	−2870	−2684
	LG3	0.018	0.036	0.028	0.220 ± 0.03	0.659 ± 0.05	−2431	−2113
	LG4	0.019	0.036	0.030	0.224 ± 0.03	0.647 ± 0.06	−2088	−1602
Sperm viability	KG	0.001	0.001	0.010	0.083 ± 0.01	0.167 ± 0.02	−2505	−2415
	WM	0.001	0.001	0.010	0.083 ± 0.01	0.167 ± 0.02	−2492	−2402
	LG2	0.001	0.001	0.010	0.083 ± 0.01	0.167 ± 0.02	−2870	−2684
	LG3	0.001	0.001	0.012	0.071 ± 0.01	0.143 ± 0.02	−2431	−2113
	LG4	0.001	0.001	0.013	0.067 ± 0.01	0.133 ± 0.02	−2088	−1602
Sperm abnormalities	KG	0.007	0.008	0.065	0.088 ± 0.01	0.188 ± 0.03	−2505	−2415
	WM	0.007	0.009	0.065	0.086 ± 0.01	0.198 ± 0.03	−2492	−2402
	LG2	0.008	0.007	0.070	0.094 ± 0.01	0.176 ± 0.03	−2870	−2684
	LG3	0.008	0.007	0.072	0.092 ± 0.01	0.172 ± 0.03	−2431	−2113
	LG4	0.009	0.007	0.075	0.099 ± 0.01	0.176 ± 0.03	−2088	−1602
Sperm concentration	KG	0.262	0.657	0.999	0.137 ± 0.02	0.479 ± 0.04	−2505	−2415
	WM	0.265	0.682	0.999	0.136 ± 0.02	0.487 ± 0.04	−2492	−2402
	LG2	0.266	0.660	0.998	0.138 ± 0.02	0.481 ± 0.04	−2870	−2684
	LG3	0.266	0.667	0.998	0.138 ± 0.02	0.483 ± 0.04	−2431	−2113
	LG4	0.266	0.669	1.043	0.134 ± 0.02	0.473 ± 0.04	−2088	−1602

KG = Koops and Grossman function, WM = Wilmink function, LG2 = second order Legendre polynomial functions, LG3 = third order Legendre polynomial functions, LG4 = fourth order Legendre polynomial function. $\sigma_{a}^{2}$ = additive genetic variance, $\sigma_{pe}^{2}$ = permanent environmental variance, $\sigma_{e}^{2}$ = error variance, $h^{2}$ = heritability, t = repeatability, −2logL = minus twice the logarithm of the likelihood, Akaike’s information criterion (AIC).

## Data Availability

Additional data are available upon request from the corresponding author.
